# Counselors' Perceptions of Their Preparedness for Telemental Health Services: A Phenomenological Examination

**DOI:** 10.1089/tmr.2021.0011

**Published:** 2023-10-04

**Authors:** Daniel C. Holland, Jeffry L. Moe, Alan M. “Woody” Schwitzer, Shana Pribesh, Jeanel Franklin

**Affiliations:** ^1^ School of Psychology & Counseling, Regent University, Virginia Beach, Virginia, USA.; ^2^ Department of Counseling & Human Services, Department of Counseling & Human Services, Old Dominion University, Norfolk, Virginia, USA.; ^3^ Department of Educational Foundations and Leadership, Department of Counseling & Human Services, Old Dominion University, Norfolk, Virginia, USA.; ^4^ Counselor Education and Supervision Doctoral Student, Department of Counseling & Human Services, Old Dominion University, Norfolk, Virginia, USA.

**Keywords:** distance counseling, technology mediated, telemental health, phenomenology, counselor education

## Abstract

**Background:**

To examine counselors' perceptions of their formal preparation for engaging in telemental health (TMH) counseling with the intent of gaining an understanding of their lived experiences.

**Materials and Methods:**

Semistructured interviews were conducted with seven seasoned counselors who regularly engage in technology-mediated distance counseling.

**Results:**

The results highlighted themes within two emerging categories: the counselor and training/education. Themes related to motivation and specific counselor attributes emerged from the first category and themes of availability, inadequacy, and modality emerged from the second category.

**Discussion:**

The implications from this study suggest a lack of availability and standardization of effective training on TMH delivery.

**Conclusion:**

This study identifies areas of potential future research related to counselors' preparation experiences as well specific areas of need for TMH training in counseling graduate programs and other natural opportunities.

## Introduction

The increase in technology availability and its application in social contexts have facilitated a global increase in technology-mediated connection, which, for many, has normalized its use for medical purposes,^1,2^ specifically mental health care.^3^ The conversational nature of psychiatric and psychotherapeutic services makes telemental health (TMH) care particularly effective and, in many cases, providing a stronger working alliance than in-person face-to-face services.^4,5^ A growing number of researchers have examined the various methods of distance therapy with an emphasis on the client's experience.^6,7^ The steady growth of technology availability and the need for distance services have left professional counselors with the task to ethically, safely, and effectively utilize new tools and systems to provide services.^8^ Many professional organizations such as the American Counseling Association (ACA), American Psychological Association, American Association of Marriage and Family Therapy, and the American Mental Health Counselors Association have embraced the challenge to prepare their disciplines.^8^

Although professional organizations created standards of care regarding TMH, providers were reluctant to implement TMH before the COVID-19 outbreak.^9^ Recent literature has largely focused on informing providers of best practices, challenges, and other professional issues that may arise when providing TMH.^10,11^

There continues to be a lack of literature on the education and training process for TMH providers that has created a gap in knowledge pertaining to how these identified best practices and standards are being met by TMH providers. Strong logistical and ethical concerns exist among many professionals and organizations within the counseling profession about TMH.^3^ Understanding how counselors experience and perceive their TMH education and training can shed light on gaps in preparation and training efforts. Addressing these training gaps in education programs, could increase counselors' comfort and familiarity with TMH, resulting in increased utilization. The void of existing research seems to indicate that the process of training counselors in technology-mediated distance counseling is not adequately understood, with a bias toward the assumption that counselors can generalize the counseling process from face-to-face methods without further preparation.^10^ Formal educational programs provide a rigor of preparation in a variety of issues including multiculturalism, ethics, counseling skills, professional standards, and other practices unique to counseling.^12^ In each of these areas, TMH carries its own unique differences from the face-to-face methods, yet training on these uniqueness's is significantly under-represented if addressed at all in formal counselor education.^9,10,13^

The focus of this phenomenological examination was based on the assumption that preparation experiences of professional counselors using TMH to provide services were varied and that giving voice to those experiences and their perceptions of those experience could assist counselor education programs to develop formal preparation processes for this delivery method. This study has explored the central research question, “What are the experiences and perceptions of professional counselors' preparation for utilization of telemental health methods?”

## Methods

### Study design

Phenomenological methods were selected to best describe the experiences and perceptions of participants. Through this study, data were collected from participant interviews and shared documents using a qualitative phenomenological methodology and were then analyzed. The codes and themes that emerged were used to synthesize a description of the experiences and perceptions of professional counselors' preparation for the utilization of TMH counseling. This study was found to be exempt from full institutional review board review by the Old Dominion University Review Board (study ID: 815701-1).

### Participants

Criterion and snowball sampling were used to identify participants who were state licensed counselors and spent 8–10 h per month utilizing TMH counseling. Participants included four females and three males, six of them identifying themselves as Caucasian or White and one identifying as African American with ages ranging from 34 to 62 years. The seven participants resided in various parts of Virginia and are licensed professional counselors (LPCs) in the Commonwealth of Virginia, with a few holding licenses in multiple states. Although all of the participants regularly engaged TMH counseling in a private practice setting, four held doctoral degrees and engaged in counselor education in various Council for the Accreditation of Counseling and Related Education Programs (CACREP) programs and one was a student in a CACREP counselor education and supervision program.

Their collective years of experience in clinical settings ranged from 6 to 39 years, with an average of 17 years and from 2 to 8 years specifically providing TMH services, with a mean of 4 years.

### Data collection

The lead researcher conducted one primary semistructured interview as the primary method of data collection to address the research question: “What are the experiences and perceptions of professional counselors' preparation for utilization of technology-mediated distance counseling methods?” To better understand and extract detailed personal explanations of their perceptions and experiences of the phenomenon, the researcher utilized attending skills to include active listening, reflection, encouragers, and other clinical listening skills. When necessary, prompts, follow-up questions, and probes were utilized to gain further clarity and a more complete understanding of their responses. Reflexive journaling through handwritten notes was utilized to record the lead researcher's personal thoughts and observations during and after the interviews. Reflexive journal notes were processed with research team members and an auditor to reduce researcher bias. The process of data collection took 3 months to complete.

### Data analysis

Each interview was recorded digitally and saved to a secured drive that was only shared with a professional transcriptionist, experienced in phenomenological research transcription. The decision to utilize a transcriptionist was made by the lead researcher in an attempt to bracket and distance himself from the data. Once documents were collected and the interviews transcribed, the data analysis process began with a review of documents, e-mail correspondence, and the transcribed interviews from participants. This provided the content for triangulation through the zigzag method.

General coding procedures included open coding, axial coding, and selective coding. Beginning with interviews 1–3, research team members conducted individual horizontalization independently, where each member of the team used the coding process by writing on the transcript, circling keywords, and making notes for other members of the team to read and share during times of collaboration. This process also assisted in the reduction of researcher bias in selective perception, which can cloud clear outcomes and data analysis. This was followed by the development of consensus codes that began to identify textural descriptions. Research team collaboration assisted with developing the first codebook (#1). This process was repeated for interviews 4—7, utilizing individual horizontalization, and then developing our consensus codes to identify our rich textural descriptions, which assisted with the development of the codebook (#2). Once finalized, the independent auditor examined the code books, textural and structural descriptions, as well as the audit trail to ensure the trustworthiness of the study. The total time for the data collection and analysis process was ∼13 weeks.

## Results

Upon completion of major thematic analysis, the study findings were organized into themes that are either related to the counselors or specific to their training/education experience (Table 1).

**Table 1.** Major Thematic Analysis

The counselor	Training/education
*Motivation (1)*	*Availability (3)*
• Client driven (1.1)	• Difficulty locating (3.1)
• Culturally driven (1.2)	• Lacks standardization (3.2)
• Counselor driven (1.3)	• Absent from graduate programs (3.3)
*Counselor attributes (2)*	*Inadequacy (4)*
• Autonomy (2.1)	• Poorly defined (4.1)
• Clinical skills (2.2)	• Lacks specificity (4.2)
• Self-awareness/boundaries (2.3)	• Ineffective at preparation (4.3)
	*Modality (5)*
	• Practicality (5.1)
	• Natural opportunities (5.2)
	• Limitations (5.3)

### The counselor

Motivation was one of the primary themes expressed by participants related to the counselor. Participant motivation was found to be primarily client driven, culturally driven, or counselor driven. All of the participants expressed a level of motivation directly related to TMH benefits for their clients. Counselor-driven motivators included the expansion of client access or the strong desire to reach a certain population. Counselor 1 described her work counseling individuals overseas who are physically limited in accessing professional counseling that created a barrier in gaining services. Counselor 2 echoed that thought regarding clients who may prefer not to be in person saying, “I think the success part of it is some people just don't want to come to your office, the stigma of that, that's really success, just getting into peoples' homes, people who would not otherwise come to counseling.”

Emerging patterns from participants identified certain attributes of the counselor that contribute to the preparation and involvement in TMH counseling that included autonomy, clinical skills, and self-awareness and boundaries. All seven participants demonstrated autonomy by individually pursing additional education related to TMH counseling and migrating training and skills designed for face-to-face counseling to their TMH work. “Training, none formally,” Counselor 2 stated adding, “informally I have had to go out to websites; I have a couple of associates who do it. I have YouTube, I have read, but formally nothing.” Although most of these skills were adapted from face-to-face formal education, some were from as Counselor 2 stated, “trial and error.” In addition, they all demonstrated a foundational understanding of self-awareness and the need for boundaries unique to TMH environments such as counselor accessibility.

### Training and education

Themes related to training and education involved its availability, inadequacy, and modality (Table 1). A theme of inadequacy was sustained by opportunities that were poorly defined, lacked specificity, and were not considered effective. The delivery or modality of training surfaced specifically related to its practicality, lack natural opportunities and limitations. Each of the seven participants reported having no access to proper or effective training within their graduate programs other than what they could migrate from their face-to-face training through “trial and error” and, after seeking out other forms of education and training, expressed frustration with the limited availability of opportunities. “None. It was never discussed,” Counselor 2 relayed when asked about his graduate level counselor education program's offering for TMH training.

Although “trial and error” seemed to be the most widely utilized method of learning from all seven participants, most of the training available through workshops and conferences seemed to lack a standardization of best practices and was presented in a more subjective manner. Participants interviews also revealed that most training received was either self-led PowerPoints, lectures, or general courses on topics (i.e., ethics) with highlights directed toward distance counseling. Counselor 3 eloquently phrased a sentiment shared by all of the participants when she said, “the workshops I attended …were not about how-to.” She reported the same practical issue that Counselors 6 and 7 mentioned in their interviews without prompting: where distance counselors should look on the screen. She stated, “the client only experiences the counselor looking at them if the counselor is looking at the camera. If the counselor actually looks at the video feed of the client, then the client experiences the counselor looking down and not looking at them.”

**FIG. 1.**
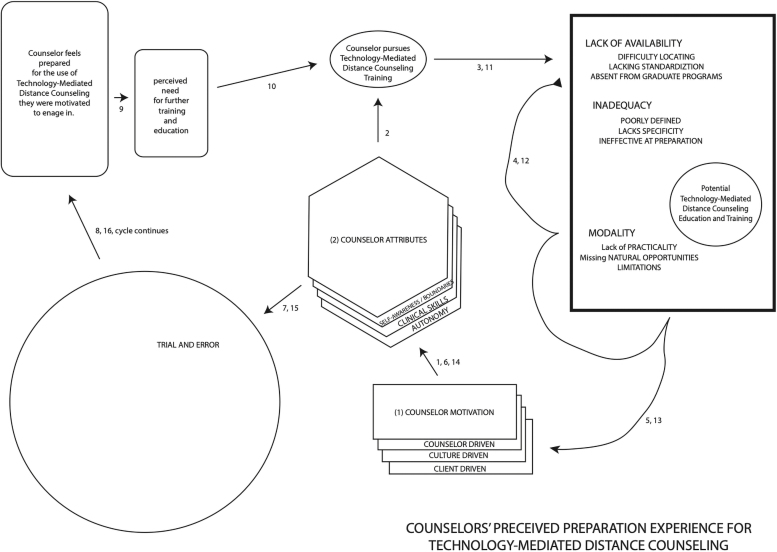
Conceptual map of research team findings.

All of the participants mentioned the utilization of the existing preparation structure in graduate school through licensure as potential natural opportunities for more effective preparation experiences. Counselor 6 recommended utilizing the natural opportunity of practical learning at a graduate level, “the same sort of thing that you would do at a group counseling class, where you participate in a group as a part of a class and do some mock video counseling.” Counselor 2 introduced a limitation stating that existing faculty may not “know how to teach it.” Lack of supervisor experience and lack of educational outlets (books, practical training, etc.) seemed to contribute to some negativity of counselors' perceptions of their preparation experience (Fig. 1).

## Discussion

Emerging themes surrounding counselor attributes give voice to the importance of a counselors' level engagement in the preparation process. Their motivation was critical for their development in the void of readily accessible training and education. As noted in the literature, most of the participants were aware of the positive attributes of the delivery method, particularly that of therapeutic alliance, ^4,6,14^ and acknowledged that they were often motivated by client need, comfort, and preferences.^15,16^ Participant autonomy provided confidence in their pursuit of education and training to gain a level of proficiency that the National Board for Certified Counselors (NBCC) describes as, “qualified by education and experience” (Standard 1). Yet these participants have pursued the education necessary to develop and demonstrate competence, through their understanding of self-awareness, boundaries, and their own clinical adaptations. All seven participants were able to clearly define their boundaries through a strong sense of self-awareness that increased their confidence in what they felt would be their most productive uses of this medium, and reduction of risk for other issues.^8,13^

Factors specific to training and education provide insight into barriers TMH counselors may experience in their pursuit of adequate preparation in their competence^13^ and compliance^6^ with various standards.^8,17,18^ Their experiences also supported the literature's implied bias toward the assumption that counselors can generalize the counseling process from face-to-face methods, without formal preparation.

Participant responses indicated an overall inadequacy of the training and education they could find, leading a majority of participants to engage in “trial and error.” All seven participants agreed that lecture-based education or self-led PowerPoints are effective ways of gaining some general knowledge, but are not the modality that they utilized to develop skill and confidence in TMH care delivery. The American Telemedicine Association states that individuals who utilize technology to perform services “shall be trained in the correct usage.”^8,19^ This research indicated that according to participants' perceptions of their preparation experience, to “be trained” would involve hands-on training, supervision, or mentorship through natural outlets such as practicums, internships, and residency opportunities, in a practical way, through professionals who have experience in these types of services. This is consistent with previous suggestions for counselor educators to incorporate modeling of TMH practices in the classroom.^9^

### Implications for graduate programs

Graduate programs should consider how both TMH education and training could be programmatically scaffolded into their curriculum. This may begin with the development of a standardized track or curriculum that is fully integrated into existing programming complete with assessment and evaluation. Special consideration should be given to the modality of training, to include practical hands-on training to augment the content material. Natural opportunities should be considered as possible avenues for training such as practicum, group classes, advanced skills classes, internship opportunities, and residencies. A method of evaluation of training effectiveness would benefit any program implementing training into their protocol. Finally, graduate programs can assist students beginning their professional journey and practice with the connection of other professionals engaging in TMH counseling, and to organizations who continue to support those professionals.

### Implications for professional organizations

This study also yields findings that have reaching implications for professional organizations to include CACREP, ACA, Association for Counselor Education and Supervision (ACES), and the NBCC. Even though most of these organizations place an emphasis on distance counseling practices and standards, more emphasis needs to be given to the provided preparation of those attempting to or actively engaging in the process of distance counseling and the effectiveness of those efforts. Finally, a stronger presence in the CACREP standards for counselor education could lead to a standardized process for programmatic scaffolding of relevant education and training experiences specific to TMH delivery standards and best practices. This would allow CACREP self-studies and sequential reviews to assist graduate counseling programs with articulating the need for and implementing a standard of training accessible to developing professional counselors within CACREP programs.

### Limitations

The research team recognized that there were various limitations to this study. Possible limitations of this study include the influence of personal biases, the selection of participants and data collection, and use of a professional transcriptionist. It was vital for researchers to be aware of all biases throughout the stages of this study. Possible implications of personal biases were explored before the study began, and methods to reduce their influence during each stage were carefully considered throughout the process. The primary researcher engaged in reflective journaling to record thoughts and concerns and debriefed these thoughts with research team members and the auditor. In addition, member checking was utilized to confirm accuracy of the transcripts while confirming emerging themes and patterns to ensure that statements gathered were free from this researcher's influence. Multiple contacts were made with research team members as well as the auditor to ensure that researcher biases were minimized.

The sample size of this study was limited with seven participants who were professional counselors from Virginia who were experienced with and actively engaging in TMH counseling. It is believed that widening the geographical range with an increased number of participants may have reduced this limitation. Homogeneous data collection is encouraged.

Interviews were differentiated between face-to-face and telephone interviews. Supporting documentation collection and additional communication were similar in content and frequency between the lead researcher and the participants. The semistructured interview process had inherent limitations regarding questioning, which the lead researcher attempted to control by making every effort to reduce deviation from the scripted interview protocol, attempting to ensure the highest level of standardization.

Participant criteria for selection were based on their level of professional experience. Participation was limited to LPCs with a minimum of a master's degree who engaged in distance counseling 6–8 h per month. As a result, demographic diversity was unintentional. Transferability of this study's findings may be limited due to these participant attributes.

### Recommendations for future research

Future research on the phenomenon of the preparation experiences of TMH counselors is needed. Expanding research into graduate-level CACREP-accredited educational programs and their role in preparation experiences for counselors would be of benefit. These findings would benefit counselor education programs, professional organizations, and their training divisions as well as private training providers to design programs that naturally integrate the balance of content that is necessary for safe practice and the practical experience of skills unique to the technology-mediated interface. This research could also provide a foundation for new standards through CACREP and other avenues of accreditation and licensing as well as a standardization of a baseline level of functional understanding for future professional counselors.

This study's findings can inform future research on counselor education programs, counselor educators, professional support organizations, clinical supervision, and counseling supervisors. Owing to the limited amount of literature related to this topic, further research involving a more diverse sampling of professional counselors who engage in TMH counseling, utilizing both qualitative and quantitative methodologies, would be recommended.

## Conclusion

This study sought to synthesize the perception of counselors' preparation experiences for the utilization of technology-mediated distance counseling methods. The findings in this study support the need for a body of research to be developed around counselors' preparation experiences for the utilization of TMH counseling. The findings of this qualitative phenomenological study have contributed to the general understanding of the preparation phenomenon on a core level, providing a foundation for future research. These findings may be instrumental in the process of informing stakeholders who are concerned with enhancing and improving the counselor preparation experience for technology-mediated distance counselors.

## Abbreviations Used

ACA: American Counseling Association
LPCs: licensed professional counselors
TMH: telemental health
